# Comparative Evaluation of Four Potent *Neospora caninum* Diagnostic Antigens Using Immunochromatographic Assay for Detection of Specific Antibody in Cattle

**DOI:** 10.3390/microorganisms9102133

**Published:** 2021-10-11

**Authors:** Ragab M. Fereig, Hanan H. Abdelbaky, Yoshifumi Nishikawa

**Affiliations:** 1 National Research Center for Protozoan Diseases, Obihiro University of Agriculture and Veterinary Medicine, Hokkaido 080-8555, Japan; ragabfereig2018@gmail.com (R.M.F.); hananragabegypt@gmail.com (H.H.A.); 2 Department of Animal Medicine, Faculty of Veterinary Medicine, South Valley University, Qena 83523, Egypt

**Keywords:** neosporosis, antigen, antibody, immunochromatography, cattle

## Abstract

*Neospora caninum* is an intracellular protozoan parasite responsible for numerous abortion outbreaks and neonatal abnormalities in cattle. Rapid and accurate diagnosis is critical for *N. caninum* control owing to the lack of vaccine or drug-based control strategies. Herein, we evaluated the performance of four frequently used antigens in the diagnosis of *N. caninum* infection using immunochromatographic tests (ICTs) as a rapid, affordable, and field applicable tool. These antigens included recombinant proteins of *N. caninum* surface antigen 1 (NcSAG1), dense granule proteins 7 (NcGRA7) and 6 (NcGRA6), in addition to native *Neospora* lysate antigen (NLA). Our study revealed the utility of all antigen-based ICTs for detection of specific antibodies to *N. caninum*. However, the NcSAG1-based ICT was the best for detection of all control *N. caninum*-infected mouse or cattle sera, while NcGRA7 and NcGRA6-based ICTs exhibited specific ability to detect samples from acute and sub-acute infection in mice and cattle, respectively. Analyses of the NcSAG1-based ICT against enzyme-linked immunosorbent assays (ELISAs) of the same antigen revealed its efficiency in detection of field cattle samples as observed in high sensitivity (84.2%), specificity (93.5%), agreement (90%), and kappa value (0.78). The current knowledge provides an efficient platform for *N. caninum* control through on-site diagnosis of infected cattle.

## 1. Introduction

Neosporosis is a protozoan parasitic disease caused by *Neospora caninum*, which has been recorded among a wide variety of animal hosts and in most countries [1]. Three developmental stages have been recorded for *N. caninum*: tachyzoite (rapidly growing stage), bradyzoite (slowly growing stage), and sporozoite (fecal oocyst). Canines act as definitive hosts while many other animals, particularly cattle, sheep, and other ungulates, are intermediate hosts. Horizontal and vertical transmissions are the common routes of infection [2,3]. *N. caninum* is responsible for abortion in cattle, resulting in drastic financial losses in the livestock industry attributable to the abortion per se, loss of milk production, and costly control measures involving treatment and culling procedures [4,5].

Today, various diagnostic techniques are available for the detection of *N. caninum* infection. In cases of *Neospora* abortion in cattle, histopathology and immunohistochemistry (IHC) using tissues from aborted fetuses are considered the definitive tests [6]. The polymerase chain reaction (PCR) is also used for the determination of parasite-specific nucleic acids in samples from aborted animals, such as brains and placenta [7]. However, the high costs, special equipment requirements, and need for skilled persons when applying IHC and/or PCR restrict their use on a large scale [8].

Serological detection using different antibodies (Immunoglobulins G and M) is frequently used for diagnosis of *N. caninum* infection in different animals. Numerous serological tests have been used against *N. caninum,* including the indirect fluorescence antibody test (IFAT), enzyme-linked immunosorbent assay (ELISA), and Western blotting. These tests are regarded as efficient diagnostic tests for *N. caninum* antibody detection either in field or experimental animals when potent and specific antigens are used [9]. In addition, the detection of specific antibodies in sera of infected animals is frequently used to detect acute, sub-acute, or chronic infection [10]. IgM and IgG-based detection are useful approaches to *Neospora* diagnosis and control because of their capability for differentiation between acute and chronic infection, respectively [10,11]. Previous studies have established surface antigen 1 (SAG1), SAG1-related sequence (SRS2), and dense granule protein 6 (GRA6) or GRA7 to be the most frequently used antigens for diagnosis of *N. caninum* infection, in either cattle or dogs [9,12]. In addition, anti-NcSAG1 antibodies have been reported in both acute and chronic *N. caninum* infection, whereas anti-NcGRA7 antibodies have been widely accepted as markers for acute *N. caninum* infection [9,13,14,15]. Moreover, the diagnostic and immunomodulatory properties of NcGRA6 have been reported [16,17]. On the other hand, the potential of *Neospora* lysate antigen (NLA) for detection of specific antibodies to *N. caninum* infection has also been reported. Therefore, many research groups are still using NLA as a standard antigen to validate newly developed antigens [9,12].

Herein, we proposed to establish a useful diagnostic tool for detection of specific antibodies against *N. caninum* infection in cattle based on the rapid immunochromatographic test (ICT). Only one study has investigated the utility of such an approach for *N. caninum* diagnosis. Liao et al. (2005) [18] found that the NcSAG1-based ICT is useful for detection of infected sera from mice, dogs, and cattle. In addition, Pinheiro et al. (2005) presented dot-ELISA as a quick serologic method for detection of anti-*N. caninum* antibodies in dogs [19]. However, since then, no other studies have been reported. Thus, the current study sought a convenient ICT by comparing various antigens, recombinant NcSAG1 (rNcSAG1), rNcGRA7, rNcGRA6, in addition to native *Neospora* lysate antigen. Our study provided novel knowledge for the utility of NcGRA7, NcGRA6, and NLA-based ICTs in the detection of sub-acute infection in cattle. Also, the superiority of the NcSAG1-based ICT was proved through the capability of antibody detection in all positive control sera from different stages of infection and various animal species (mice: 2, 4, and 8 weeks post-infection (wpi); cattle: 4 and 8 wpi). This study is a great step toward the efficient diagnosis and control of *N. caninum* in cattle because it offers various potent ICTs for rapid and on-site detection of infected cattle in the field. Nevertheless, a higher number of control samples from *N. caninum*- and closely related pathogen-infected cattle will be required for further investigations and validations of our developed ICTs in the future.

## 2. Materials and Methods

### 2.1. Ethics

Stipulations and guides for the use and care of laboratory animals of the Ministry of Education, Culture, Sports, Science and Technology, Japan, in all experimental works conducted in this study were followed strictly. The procedures were approved by the Committee on the Ethics of Animal Experiments at the Obihiro University of Agriculture and Veterinary Medicine (permission numbers 18–44 (April, 2018), 19–3 (April, 2019), 19–128 (April, 2019)). All efforts were made to alleviate the animal suffering. Blood samples were collected from the heart after exposure of mice to general anesthesia by isoflurane, followed by euthanizing by cervical dislocation. Consistently, blood was collected from the heart of rabbits kept under general anesthesia after intravenous injection of sodium thiopental, followed by cervical dislocation.

### 2.2. Experimental Mice and Rabbits

Female BALB/c mice aged 6–7 weeks were purchased from CLEA Japan (Tokyo, Japan) for preparation of control sera. Female white Japanese rabbits (15 weeks old, female 3 kg) were obtained from Kitayama Labes, Nagano, Japan, for the preparation of polyclonal antibodies against recombinant proteins. Mice and rabbits were reared in the animal facility of the National Research Center for Protozoan Diseases at Obihiro University of Agriculture and Veterinary Medicine, Obihiro, Japan. Housing of the mice was under specific-pathogen-free conditions in cages. Rabbits used in this study were reared and cared for in accordance with the guidelines. The rabbits were housed in a room with a temperature of 25 °C, humidity of 40%, and controlled lighting (i.e., period of light from 6:00 to 19:00 h). The rabbits had access to tap water and commercial pellets (CR-3; CLEA, Japan, Tokyo) ad libitum throughout the experiments.

### 2.3. Parasites and Cell Cultures

Tachyzoites of the Nc-1 isolate of *N. caninum* and PRU and PLK strains of *Toxoplasma gondii* were maintained in African green monkey kidney epithelial cells (Vero cells) as previously described [17]. Finally, the parasite pellet was suspended in Roswell Park Memorial Institute (RPMI)-1640 medium (Sigma, St. Louis, MO, USA).

### 2.4. Recombinant and Lysate Antigens Preparation

Recombinant antigens of NcSAG1, NcGRA7, and NcGRA6 fused with glutathione-*S*-transferase (GST) were employed in order to detect specific antibodies of *N. caninum* in serum samples. rNcSAG1, rNcGRA7, and rNcGRA6 antigens were prepared as formerly described [17,20,21]. In addition, GST alone was used as a control antigen in all experiments to verify the obtained results for NcSAG1, NcGRA7, and NcGRA6. Expression of such antigens was conducted using the *Escherichia coli* expression system. After purification and dialysis of four antigens, NcSAG1, NcGRA7, NcGRA6, and GST, their quantity and purity were evaluated by sodium dodecyl sulfate polyacrylamide gel electrophoresis (SDS-PAGE) followed by Coomassie Brilliant Blue staining (MP Biomedicals Inc., Illkirch-Graffenstaden, France) (Figure S1). Purified tachyzoites of *N. caninum* (Nc-1) or *T. gondii* (PLK) were used for preparation of a soluble lysate antigen as described previously [22,23]. Bicinchoninic acid (BCA) protein assay kit (Thermo Fisher Scientific, Waltham, MA, USA) was used for measurements of the recombinant or lysate protein concentrations.

### 2.5. Experimental Sample from Mice

For mouse experimental samples, a total of 20 mice were divided into four groups: non-infected, 2 wpi (acute), 4 wpi (sub-acute), and 8 wpi (chronic), with 5 mice for each group. Each mouse was infected intraperitoneally via a non-lethal dose of Nc-1 tachyzoites (1 × 10^5^) prepared in 400 µL of RPMI-1640 medium. Serum samples from *T. gondii*-infected mice were prepared by intraperitoneal infection of female ICR mice by PRU (5 × 10^4^ tachyzoites/mouse). Serum was harvested after collection of blood via cardiac puncture from each mouse and addition to the tube without anticoagulants.

### 2.6. Cattle Experimental and Field Samples

Regarding experimental samples, serum samples collected from male Holstein calves aged 2–4 months at −13 (non-infected; *n* = 6), 29 days (sub-acute; *n* = 4), and 56 days (chronic; *n* = 5) after intravenous infection with 1 × 10^7^ tachyzoites of *N. caninum* Nc-1 strain were used. The experimentally infected cattle sera were confirmed using a commercial immunofluorescent antibody test slide (VMRD, Pullman, WA, USA) and an ELISA based on recombinant NcSAG1 [21,24].

Field cattle sera (*n* = 53) were collected from dams in the Nemuro subprefecture of Japan from 2007 to 2009. Among such samples, 8 out 53 were confirmed as cases of neosporosis in which *N. caninum* antigens were detected in aborted fetal tissues using IHC by the Livestock Hygiene Service Center in Hokkaido. Moreover, their mother’s sera were tested for seropositivity to *N. caninum* by IFAT (VMRD).

All efforts were made to reduce animal suffering. Experimentally infected cattle were euthanized by exsanguination under deep anesthesia as reported in detail in our previously published paper [24]. Blood collection from live experimental or field cattle was performed by expert persons or veterinarians.

### 2.7. Preparation of Polyclonal Antibodies in Rabbits

Polyclonal antibodies against NcGRA7-GST were prepared as described previously [25]. Polyclonal IgG antibodies to NcSAG1-GST and NcGRA6-GST were generated and purified as follows. The recombinant proteins (0.5 mg) were prepared in phosphate buffered saline (PBS) and emulsified in an equal amount of TiterMax Gold adjuvant (Funakoshi, Tokyo, Japan), and subcutaneously injected at multiple sites into female Japanese white rabbits on day 0. Then, an immunization regimen using the same protein adjuvant combinations was applied to each rabbit at days 14, 28, and 42 after the first immunization. For antibody monitoring, sera were collected from immunized rabbits at day −2, 12, 26, and 40 from the ear vein. After confirmation of a high antibody titer increase, blood was withdrawn from the heart at day 49. Serum at day −2 was used as a negative control samples. IgG titers were estimated by indirect ELISA using the method described below. A quantity of 2 mL of harvested rabbit serum was processed for IgG purification employing protein A chromatography columns (Bio-Rad Laboratories, Hercules, CA, USA). Qualitative analyses concerning the purity and quantity of purified IgGs were assessed by SDS-PAGE (Figure S1), and BCA protein assay kit was used for measurement of different IgG concentrations.

### 2.8. Indirect ELISA

ELISA procedures were performed as previously reported [8], with some adjustments. ELISA 96-well plates were coated with different recombinant antigens, NcSAG1, NcGRA6, rNcGRA7, or GST, at final concentrations of 0.1 μM and native antigens (NLA or TLA) at 5 μg/mL. Plates sensitized with each antigen were reacted against the serum samples diluted with PBS containing 3% skimmed milk (PBS-SM) at 1:500 and 1:200 for IgG and IgM, respectively, in mice, and at 1:300 for both IgG and IgM in cattle. In cases of secondary antibody, the plates were treated with horseradish peroxidase-conjugated anti-mouse or anti-bovine IgG or IgM, diluted at 1:6,000 and 1:4,000 for mouse IgG and IgM, respectively, or at 1:10,000 for cattle IgG and IgM. In case of naturally infected cattle sera, the cutoff point of the NcSAG1-based ELISA was calculated as the mean A415 value for standard *N. caninum*-negative sera (*n* = 6) plus 10 standard deviations (IgG = 0.132; IgM = 0.020).

### 2.9. ICT

**Figure 1:**
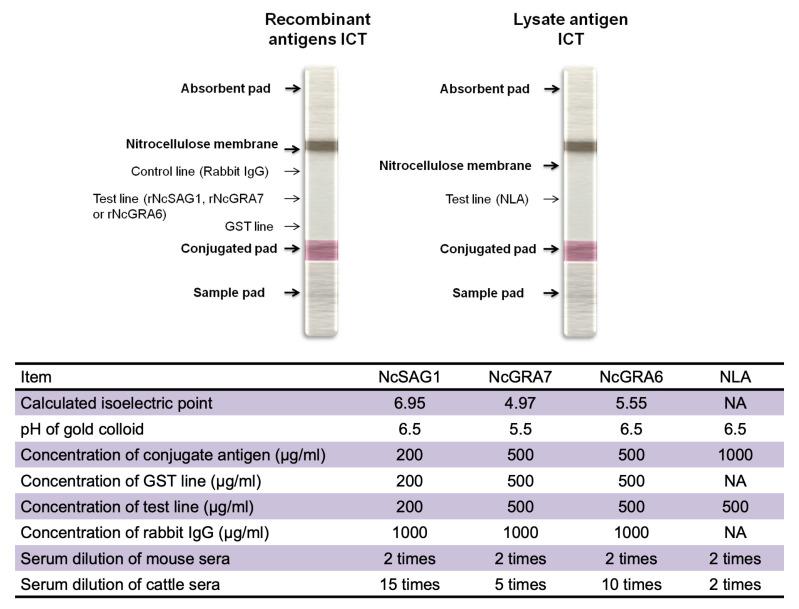
Immunochromatographic test (ICT) design and preparation. (Upper panel) Schematic diagram for the ICT prepared from recombinant antigens (NcSAG1, NcGRA7, or NcGRA6) or native lysate antigen from *N. caninum* tachyzoite (NLA). Three lines were sprayed onto the nitrocellulose membrane, including two control lines containing recombinant GST-tagged protein or anti-rabbit IgG and one test line containing recombinant antigen. While in the case of NLA-based ICT, only a line representing native antigen was prepared. (Lower panel) A table summarizing the used antigens concentrations and serum dilutions.

Preparation of gold-antigen conjugates was performed by mixing rNcGRA7-GST or rNcGRA6-GST at a concentration of 0.5 mg/mL with a gold colloid (British Biocell International, Cardiff, UK) (1:10, vol/vol) at pH 5.5 or 6.5, respectively, and incubation at room temperature for 20 min. In the case of NcSAG1-GST, the ICT was prepared as described previously [18], with some modifications. Conjugation of NcSAG1 with the gold colloid was applied at a concentration of 0.2 mg/mL at pH 6.5. In the case of NLA, conjugation with gold colloid was applied at a concentration of 1 mg/mL at pH 6.5. Thereafter, the conjugate particles were stabilized and blocked using a mixture of 0.05% polyethylene glycol 20,000 (PEG) and 1% bovine serum albumin (BSA). After centrifugation at 18,000× *g* for 30 min, approximately 90% of the supernatant was discarded by aspiration to avoid pellet disturbances. The pellet was then resuspended by brief and gentle sonication, washed with PBS containing 0.05% PEG and 0.5% BSA, and re-centrifuged as previously stated. The pellet was then diluted in 5% sucrose prepared in 10 mM Tris-HCl (pH 8.2) at 10 times less than the original volume, sprayed onto glass fiber (Schleicher & Schuell BioScience, Inc., Keene, NH, USA), and then dried at room temperature (RT) overnight in a dry and dark place. Rabbit IgGs generated against rNcSAG1-GST, rNcGRA7-GST, or rNcGRA6-GST were purified with an Econo-Pac protein A kit (Bio-Rad Laboratories). Using PBS as diluents, purified antigens (NcSAG1-GST, 0.2 mg/mL; NcGRA7-GST, 0.5 mg/mL; NcGRA6-GST, 0.5 mg/mL; or GST alone, 0.2–0.5 mg/mL) and 1 mg/mL rabbit IgG were linearly jetted onto nitrocellulose (Schleicher & Schuell) with a BioJet Quanti 3050 quanti-dispenser (BioDot Inc., Irvine, CA, USA). In the case of NLA, only a test line using lysate antigen at a concentration 0.5 mg/mL was sprayed on the membrane. The nitrocellulose membranes were left at room temperature for 2 h for desiccation, followed by blocking by a solution of 0.5% casein in 50 mM boric acid buffer, pH 8.5 for 45 min. Then the membranes were washed in a solution of 50 mM Tris-HCl (pH 7.4) containing 0.05% sodium cholate and 0.5% sucrose for another 45 min. Finally, the membranes were desiccated by incubation in a dry, dark, and ambient place overnight. Then ICT strips were prepared by arrangement of a nitrocellulose membrane, absorbent pad, conjugate pad, and sample pad onto an adhesive card (Schleicher & Schuell) and cut using a BioDot cutter (BioDot Inc) into 3-mm-wide strips. For sample testing, 35 μL of diluted serum in PBS (vol/vol) as shown in Figure 1 was placed on the sample pad by pipetting. The development of band coloration was judged within 20 min for test or control lines. To optimize the reactions on the ICT, antigen concentrations, serum dilutions, and gold colloidal pHs were tested. More details of the ICT designation and preparation are shown in Figure 1.

### 2.10. Statistical Analyses

Data analyses and calculation were performed by GraphPad Prism 5 software (GraphPad Software Inc., La Jolla, CA, USA). Statistically significant differences in the optical density values of the ELISA were estimated and interpreted using a two- or one-way analysis of variance (ANOVA) and by the Tukey–Kramer test for comparing different groups. The online statistical tool (www.vassarstats.net, access on 6 June 2020) was used for estimations of agreement proportion, kappa values, specificity, sensitivity, and 95% confidence intervals. The kappa value was categorized as fair (0.21–0.40), moderate (0.41–0.60), and substantial (>0.61) for judgment of agreement strength. Pearson’s correlation coefficient was applied to test the correlation between relative intensity in the ICT band and the ELISA absorbance values. To calculate the relative intensity of the ICT band, ICT pictures were converted into 8-bit JPEG images, then the intensity of the gray scale images was analyzed using ImageJ software v. 1.49 (Windows version of NIH Image, http//rsb.info.nih.gov/nih-image/, access on 8 June 2020).

## 3. Results and Discussion

### 3.1. First Assessment of Various Antigen-Based ICTs Using Experimental Mouse Sera

Efficient diagnosis is necessary for *N. caninum* control in cattle because of the lack of potent vaccines and effective drugs. Serological diagnosis via detecting specific antibodies has been reported as the most reliable tool for diagnosis of *N. caninum*. The diagnostic potentials of NcSAG1, NcGRA7, NcGRA6, and NLA have been reported using various immunodiagnostic approaches [9,12]. Recently, an efficient serodiagnostic approach based on the ELISA from chimeric antigens has been introduced to the field of *Neospora* control [26]. However, in the current study we attempted to develop potent ICTs by comparing various antigens, recombinant NcSAG1, rNcGRA7, rNcGRA6, in addition to native *Neospora* lysate antigen. Based on previous reports, anti-NcGRA7 antibodies appear to be specific for early *N. caninum* infection in cattle, dog, and mouse sera, whereas anti-NcSAG1 antibodies are persistent and can be detected during acute, sub-acute, or chronic *N. caninum* infection [9,13,14,15]. In addition, NLA and NcGRA6-based ICTs were developed because many previous reports had confirmed the diagnostic potentials of the two antigens [9].

**Figure 2:**
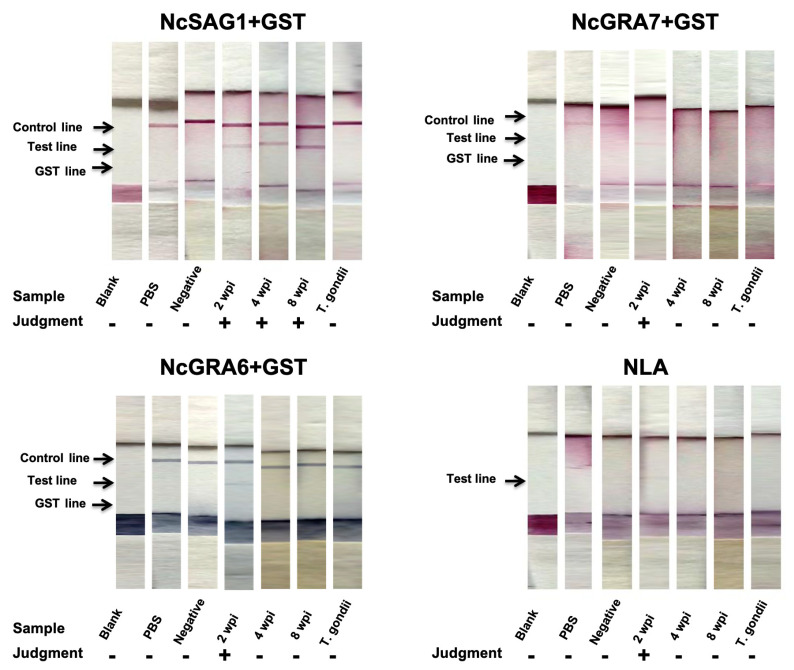
Immunochromatographic test (ICT) assay results against experimental mouse sera. A representative strip for each group of samples is illustrated for tested antigens. Blank, no treatment (*n* = 1); PBS, treated by PBS (*n* = 1); Negative (−), non-infected mouse sera (*n* = 5); positive (+), 2 wpi, mouse serum samples at 2 weeks post-infection (*n* = 5); 4 wpi, mouse serum samples at 4 weeks post-infection (*n* = 5); 8 wpi, mouse serum samples at 8 weeks post-infection (*n* = 5); *T. gondii*, sera from *T. gondii*-infected mice (*n* = 3). “−” or ”+” on graph indicates no or appearance of test band reactivity of the strip.

For efficient comparison, developed ICTs were initially assessed against control mouse sera representing non-infected and different phases of infection with Nc-1. Our novel developed ICTs for NcGRA7, NcGRA6, and NLA showed as good performance in the detection of early infection in mice as those detected by relevant ELISAs (2 wpi; NcGRA7, 5/5; NcGRA6, 4/5; NLA, 4/5) (Figure 2 and Table 1).

**Figure 3:**
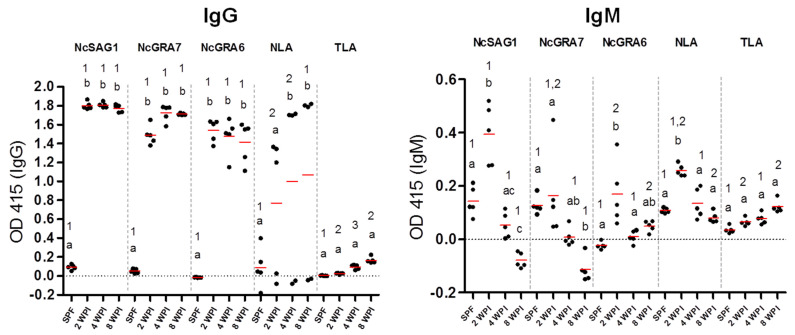
Reactivity of enzyme-linked immunosorbent assay (ELISA) against experimental mouse sera. Responses of recombinant and lysate antigens (NcSAG1, NcGRA7, NcGRA6, and NLA) were tested against mouse sera using the IgG-ELISA (**left** panel) and IgM ELISA (**right** panel). Sera from non-infected (*n* = 5) and *N. caninum*-infected (*n* = 5) for each type of sample representing 2, 4, and 8 weeks post-infection (wpi). Same samples were also tested against *T. gondii* lysate to exclude the cross-reactivity. Each bar represents the mean ± standard deviation (*n* = 5 for all groups). The different letters above the bars in the graphs indicate statistically significant differences of different sera against the same tested antigen. The different numbers above the bars indicate the statistically significant differences for the same serum sample against different tested antigens (one-way ANOVA with Tukey–Kramer post hoc analysis, *p* < 0.05).

Interestingly, this efficacy was abrogated at the later stages (4 and 8 wpi) for NcGRA7 and NcGRA6 while the antibody levels were detectable by the IgG-ELISAs. In the case of NcSAG1-based ICT, it detected all kinds of sera from infected mice at 2, 4, and 8 wpi. In the same context, the performance of NLA-based ICT was modest among tested antigens with a higher ability to detect samples from earlier stages of infection with *N. caninum* (2 wpi; 4/5, 4 wpi; 3/5, 8 wpi; 1/5). No reactivity was recorded in any of our ICTs for non-infected or *T. gondii*-infected sera, indicating their utility for detection of *N. caninum* infection without cross-reactivity against the closely related parasite *T. gondii*. Interestingly, all antigens performed well using the ELISA against tested mouse sera by discriminating between sera from non-infected (0/5) and *N. caninum*-infected mice of different stages; acute, sub-acute, and chronic infections (15/15); and *T. gondii*-infected mice (0/3). It was noteworthy that no marked differences in IgG levels against tested antigens (NcSAG1, NcGRA7, and NcGRA6) using the ELISA were observed at 2, 4 and 8 wpi (Figure 3). On the other hand, IgM levels against NcSAG1, NcGRA7, NcGRA6, and NLA were significantly higher for sera collected from acutely infected mice (2 wpi) compared with those collected from 4 or 8 wpi or from non-infected mice (Figure 3 and Table 1).

This result might be attributable to the normal antibody dynamics of IgM by increment at 7 days post-infection (dpi) followed by gradual decrease. For analyses of this result, the correlation between relative intensity in the ICT band and absorbance values in the ELISA were calculated using Pearson’s correlation coefficient. According to our observations, the specific reactivity of NcGRA7- and NcGRA6-based ICTs was predominantly observed against samples of 2 wpi, the stage at which IgM level is usually high. Thus, we estimated the correlation coefficient in sera from the early stage of infection comprising acute (2 wpi) and sub-acute (4 wpi) mouse groups (*n* = 10) (Figure S2). The results revealed strong correlation of IgM-ELISA and ICT band intensities only in NcGRA6 (Pearson’s r = 0.8580) followed by NcGRA7 (Pearson’s r = 0.6328), which might be explained by the specific reactivity of NcGRA7- and NcGRA6-based ICTs against samples of 2 wpi. In our previous studies of the ICT for other apicomplexan parasites, we also revealed the strong correlation between the reactivities of antibody isotype in the ELISA and band intensities in ICT. The *Toxoplasma gondii* dense granule protein 7-based ICT showed higher concordance with the IgG-ELISA than that of IgM [27]. In another study on *Cryptosporidium parvum* using the glycoprotein P23-based ICT, the correlation was higher in the case of IgM than of IgG [28].

### 3.2. Evaluation of Various Antigen-Based ICTs against Experimental and Control Cattle Sera

**Figure 4:**
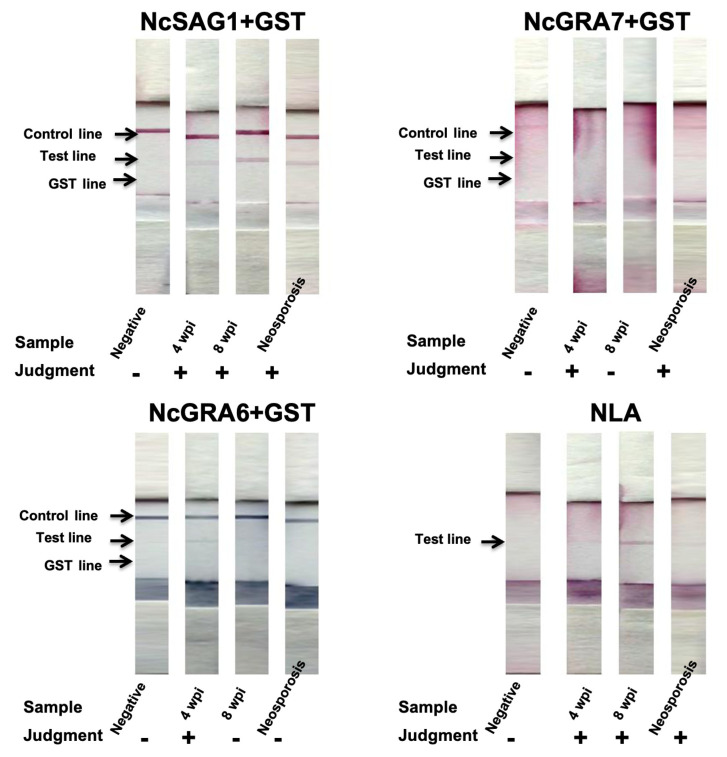
Immunochromatographic test (ICT) assay results against experimental and control cattle sera. A representative strip for each group of samples is illustrated for tested antigens. Negative, non-infected cattle sera (*n* = 6); 4 wpi, cattle serum samples from experimentally infected calves at 4 weeks post-infection (*n* = 4); 8 wpi, cattle serum samples from experimentally infected calves at 8 weeks post-infection (*n* = 5); neosporosis, sera from *N. caninum*-naturally infected heifers experienced abortion and confirmed by immunohistochemistry (*n* = 8). “−” or ”+” on graph indicates no or appearance of test band reactivity of the strip.

In the case of cattle, we compared our developed ICTs against several categories of serum samples, including experimental samples from non-infected (*n* = 6) and *N. caninum*-infected cattle (4 wpi; *n* = 4, 8 wpi; *n* = 5). In addition, samples collected from cows (*n* = 8) confirmed by a commercial IFAT for antibody detection in maternal sera and with IHC for antigen detection from aborted fetuses at the time of abortion were also considered as control-positive samples and diagnosed as neosporosis. Interestingly, NcGRA7- and NcGRA6-based ICTs showed a similar tendency in mouse assessment; such antigens showed only high reactivity against sub-acute infection (4 wpi; 3/4 for both antigen-ICTs) but no reactivity was observed in chronic infection samples (8 wpi; 0/5 for both antigens) (Table 1 and Figure 4). However, the NcGRA7-based ICT exhibited better reactivity than the NcGRA6-based ICT against samples of neosporosis (5/8 vs. 0/8). Regarding the NcSAG1-based ICT, excellent performance was observed against all positive samples, either experimental or naturally infected control cattle samples. It detected 17/17 of all infected samples (experimental 4 wpi, 4/4; experimental 8 wpi, 5/5; control-positive 8/8), without any reactivity against non-infected samples (0/6) (Table 1). In addition, we showed the usefulness of the NLA-based ICT in the detection of specific antibodies against samples of neosporosis, which were identical to those obtained in the NcSAG1-based ICT (Table 1 and Figure 4). Concerning the IgG- and/or IgM-ELISA results in cattle, NcSAG1- and NLA-based ELISAs detected all samples from different stages of infection (17/17), while the NcGRA7-based ELISA was able to detect samples of sub-acute (4/4) and of chronically infected cattle (3/5). Consistently, the NcGRA7-based ELISA demonstrated high ability to detect samples of neosporosis (6/8). In the case of the NcGRA6-based ELISA, the reactivity was also related to the earlier stage of infection (4 wpi, 2/4) with no reactivity to chronic infection (8 wpi, 0/5), as noticed also in the case of the NcGRA6-based ICT. Concerning samples of neosporosis, the NcGRA6-based ELISA detected five out of eight samples (Table 1).

**Figure 5:**
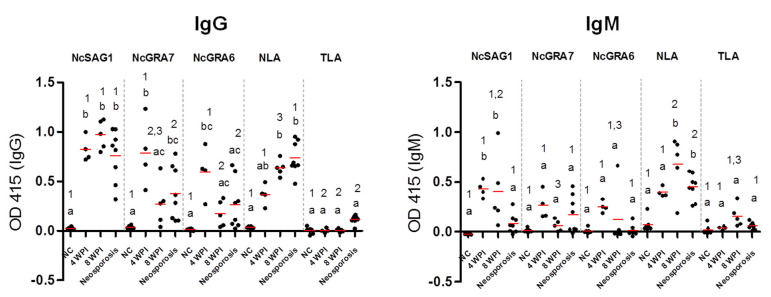
Enzyme-linked immunosorbent assay (ELISA) results against experimental and control cattle sera. Reactivity of ELISAs sensitized by recombinant and lysate antigens (NcSAG1, NcGRA7, NcGRA6, and NLA) were tested against cattle sera using the IgG-ELISA (left panel) and IgM ELISA (right panel). Sera from non-infected (*n* = 6) and *N. caninum*-experimentally infected cattle sera at 4 weeks post-infection (wpi) (*n* = 4), 8 wpi (*n* = 8), and naturally infected cattle sera termed as neosporosis confirmed by immunohistochemistry (*n* = 8). The same sera were tested against *T. gondii* lysate (TLA) to assay the cross-reactivity. Each bar represents the mean ± standard deviation. The different letters above the bars in the graphs indicate statistically significant differences of different sera against the same tested antigen. The different numbers above the bars indicate the statistically significant differences for the same serum sample against different tested antigens (one-way ANOVA with Tukey–Kramer post hoc analysis, *p* < 0.05).

Furthermore, the antibody levels of different cattle sera against all tested antigens were compared for the IgG and IgM ELISAs (Figure 5). No significant differences in IgG levels against NcSAG1 and NLA were observed among experimental samples of sub-acute (4 wpi) or chronic infection (8 wpi) or even samples of neosporosis from field cattle, while NcGRA7- and, to a lesser extent, the NcGRA6-based IgG ELISAs showed significantly higher IgG levels for sub-acute than for chronically infected cattle. For the IgM-ELISA, variations in antibody levels among samples of different stages of infection were only significant in the cases of 4 and 8 wpi for the NcSAG1-based ELISA, and 8 wpi and neosporosis for the NLA-based ELISA against sera from non-infected cattle. Nevertheless, high IgM levels of the NcGRA7- and NcGRA6-based ELISAs for sera from sub-acute infection (4 wpi) were recorded, although they were not statistically significant against sera from non-infected cattle.

**Figure 6:**
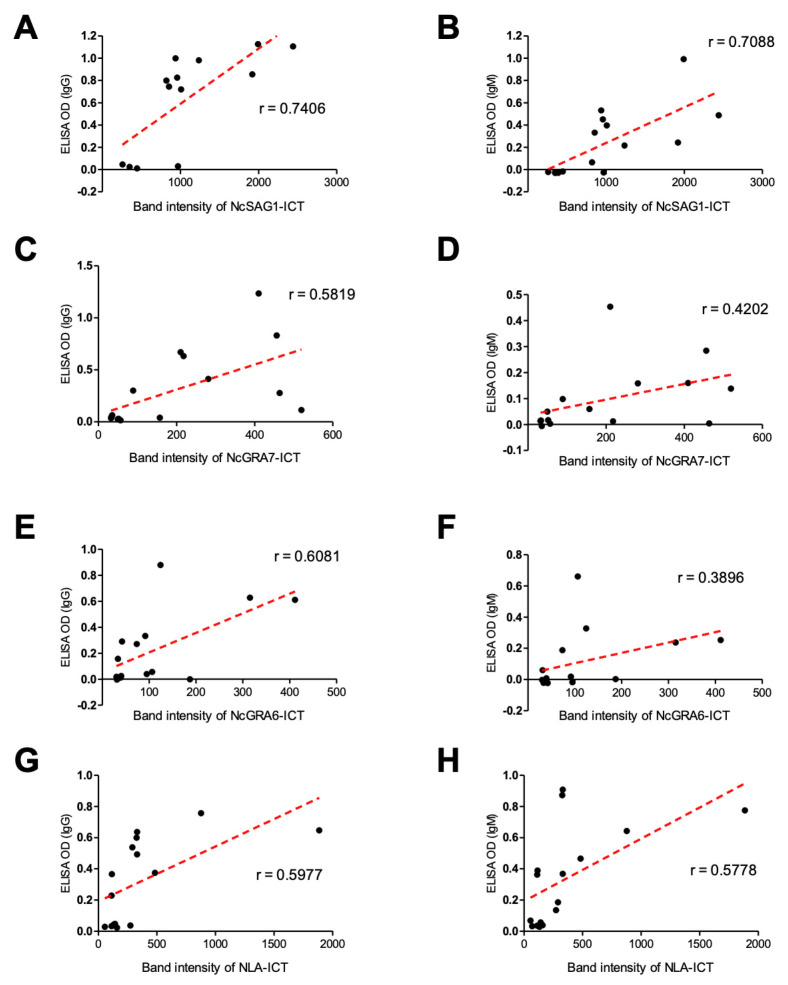
Correlation between immunochromatographic test (ICT) band intensity and enzyme-linked immunosorbent assay (ELISA) OD values of experimental cattle sera. Scatter graphs show the correlation between relative band intensity in the ICT and absorbance values in the ELISA using serum samples from non-infected (*n* = 6) and *N. caninum*-experimentally infected cattle sera (*n* = 9). The equation represents the approximation formula. The break line represents the calculated line of best fit. Correlation coefficients were calculated using Pearson’s correlation coefficient: |r| = 0.70, strong correlation; 0.5 < |r| < 0.7, moderately strong correlation; and |r| = 0.3–0.5 weak-to-moderate correlation. NcSAG1, correlation coefficient (r): r = 0.7406 for IgG-ELISA (**A**), and r = 0.7088 for IgM-ELISA (**B**). NcGRA7, r = 0.5819 for IgG-ELISA (**C**), and r = 0.4202 for IgM-ELISA (**D**). NcGRA6, r = 0.6081 for IgG-ELISA (**E**), and r = 0.3896 for IgM-ELISA (**F**). NLA, r = 0.5977 for IgG-ELISA (**G**), and r = 0.5778 for IgM-ELISA (**H**).

For better understanding of these results, the correlation coefficient was also analyzed between the band intensity of the ICT and absorbance value of the ELISA for each antigen. The high relevance of ICT results was observed in both IgG- and IgM-ELISAs (Figure 6 and Table 1). Pearson’s correlation coefficient was used to investigate the association between the relative intensity in the ICT band and absorbance values in the ELISA in experimental cattle sera (Figure 6). Best correlation was observed in case of NcSAG1 (Pearson’s r = 0.7406 and 0.7088 for IgG and IgM, respectively) followed by NLA (Pearson’s r = 0.5977 and 0.5778 for IgG and IgM, respectively). Even NcGRA7 and NcGRA6 showed a feasible correlation in both IgG and IgM (NcGRA7, Pearson’s r = 0.5819 and 0.4202; NcGRA6, Pearson’s r = 0.6081 and 0.3896 for IgG and IgM, respectively).

Slight variations in ICT response against mouse and cattle sera based on differences of antibody binding affinity are rational if we considered the animal species as a determining factor. This variation is mostly ascribed to the genetic makeup of the major histocompatibility complex (MHC), which not only induces variations among animal species but also within the same species [29,30]. Similar tendencies for NcGRA7 and NcGRA6, but not NcSAG1, in diagnostic properties in both mouse and cattle sera might be related to sharing antigen characteristics owing to the same secretory organelles.

Concerning quantitative analyses, such data represent the superiority of NcSAG1- and NLA-based ICTs over other ICTs for NcGRA7 and NcGRA6 because of the detection of a higher number of positive samples in cattle. However, the current data for developed novel ICTs using NcGRA7 and NcGRA6 provide significant prospects of discrimination between early and late infection. Such data can be exploited in the detection of neosporosis rather than chronic infection or only seropositive cases. This approach will be important in controlling neosporosis at the level of cattle farms because of the efficacy of ICTs for on-site diagnosis [4,9,12]. Furthermore, our data regarding the ICT developed from NLA as a native antigen may also have additional applications in anti-*N. caninum* antibody diagnosis.The advantages of the NLA-based ICT as a cost-effective and easily prepared product might at least facilitate the diagnosis of neosporosis in the field when recombinant antigens and antibodies facilities are not available.

### 3.3. Evaluation and Analyses of the NcSAG1-Based ICT against Field Sera from Cattle

Based on the current data, NcSAG1-based ICT and ELISA results proved its efficacy in detection of various stages of infection in mice and cattle with minimum possibility of cross-reactivity. Although the NLA-based ICT showed reactivity similar to the NcSAG1-based ICT against cattle sera, many previous reports have indicated its liability to cross-reactivity, particularly with the closely related parasite *T. gondii* [9,12]. Thus, in our subsequent experiments, we focused on the NcSAG1-based ICT for further evaluation and analyses in order to develop an ICT for efficient *N. caninum* diagnosis using antibodies. Field sera from cattle (*n* = 53) were investigated against the NcSAG1-based ICT. The previously validated NcSAG1-based ELISA [9] was then used for evaluation and verification of the NcSAG1-based ICT results. The results demonstrated that 21/53 (39.6%, CI (95%) 26.76–53.98) of cattle sera were reactive to the NcSAG1-based ICT. This result was comparable to those observed for positive samples for either the IgG- or IgM-ELISA of cattle sera (19/53) (35.8%, CI (95%) 23.5–50.25) (Table 2).

In addition, a higher seropositive rate was recorded for the IgG-ELISA (19/53) (35.8%, CI (95%) 23.5–50.25) than the IgM-ELISA (5/53) (9.4%, CI (95%) 3.52–21.42) of the NcSAG1 antigen. This result might be related to the stage of infection in animals and not to the efficiency of the NcSAG1 antigen. Thus, NcSAG1 is considered to be a broad diagnostic antigen as reported in current and previous studies [9,13,14,15].

**Figure 7:**
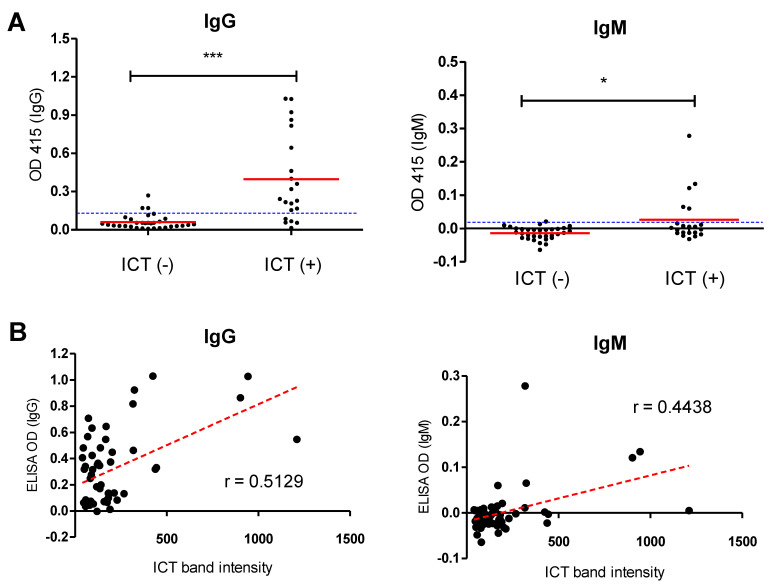
Application of the NcSAG1-based immunochromatographic test (ICT) against the enzyme-linked immunosorbent assay (ELISA) using sera from field cattle. Serum samples (*n* = 53) collected from a cattle farm were tested using ICTs for different antigens (NcSAG1, NcGRA7, NcGRA6, and NLA) and compared against IgG- and IgM-ELISAs of relevant antigens. (**A**) Comparison of the NcSAG1-based ICT and ELISA for detection of *N. caninum*-specific antibodies in field cattle. Dotted blue horizontal lines represent ELISA cutoff values. ELISA results were considered positive at an OD415 > 0.132 for NcSAG1 IgG-ELISA, and >0.020 for NcSAG1 IgM-ELISA. * *p* < 0.05, *** *p* < 0.0001, Mann–Whitney test. (**B**) Pearson’s correlation coefficient of the ICT results against different antibodies and ELISA OD values of field cattle sera. Scatter graphs show the correlation between relative band intensity in the ICT and absorbance values in the ELISA. Correlation coefficients were calculated using Pearson’s correlation coefficient: |r| = 0.70, strong correlation; 0.5 < |r| < 0.7, moderately strong correlation; and |r| = 0.3–0.5 weak-to-moderate correlation. NcSAG1, correlation coefficient (r): r = 0.5129 for IgG-ELISA, and r = 0.4438 for IgM-ELISA.

Furthermore, the NcSAG1-based ICT was further analyzed by comparing its performance against a previously validated ELISA of the same antigen. Field serum samples obtained from cattle (*n* = 53) were checked using the NcSAG1-based ICTs and compared against antibody levels of IgG- and IgM-ELISAs. The results showed a significantly higher level of antibodies (IgG or IgM) in ICT-positive samples in relation to those that were negative to ICT, which authenticated the ICT results (Figure 7). Even in a previous report using the NcSAG1-based ICT, a similar result was obtained using the IgG-ELISA [18], although the role of IgM was not analyzed. Next, we applied an objective evaluation to estimate the correlation between ICT band intensity and ELISA OD values (Figure 7). A moderately strong and a weak-to-moderate correlation for NcSAG1 was observed between the ICT and IgG- and IgM-ELISAs (Pearson’s r = 0.5129 and 0.4438, respectively).

Moreover, as shown in Table S1, sensitivity, specificity, kappa value, and agreement proportion of our developed ICT were evaluated and compared with previously validated ELISAs for the same antigen. The NcSAG1-ICT demonstrated substantial concordance with the IgG-ELISA and IgG- and/or IgM-ELISA results, as evidenced by kappa values of 0.8044, 0.7856, and 0.6376, respectively, whereas the NcSAG1-ICT showed fair concordance with the IgM-ELISA results (kappa value = 0.2293). Overall, the results in Figure 7 and Table S1 indicate the higher correlation of the NcSAG1-based ICT with the IgG but not the IgM development in infected cattle.

This result indicated the high efficacy of the NcSAG1-based ICT for anti-*N. caninum* antibody detection from field samples. Thus, this efficient diagnostic tool can be useful for *N. caninum* control if followed immediately by proper control measures, such as isolation of infected animals, quarantine measures, sanitary disposal of infected secretion, excretion, placenta, and aborted fetus. In addition, this study also suggests the usefulness of the NcSAG1-based ICT for use in epidemiological and surveillance studies based on antibody detection. This approach can also assist in establishment of control policies against *N. caninum* infection in cattle. Recognition of latent infection via detection of a specific antibody in cattle will allow the culling of the seropositive animals from the reproductive management system in the cattle farm. However, a standardized panel for the cross-reactivity assay is needed for efficient validation of the NcSAG1-based ICT before licensing for market use. This panel should include higher numbers and varieties of serum samples from cattle infected with closely related parasites such as *Besnoitia besnoiti* and *Sarcocystis* spp. or other infectious agents with similar clinical forms, such as *Brucella* spp. or *Leptospira* spp. (26). Regarding NcGRA6, NcGRA7, and NLA-based ICTs, in addition to cross-reactivity issues, further studies are required for better assessment using a higher number of control samples from different animal species and stages of *N. caninum* infection.

## 4. Conclusions

In the current study, we developed several ICTs based on potential diagnostic antigens of *N. caninum* (rNcSAG1, rNcGRA7, rNcGRA6, and native NLA). The NcSAG1-based ICT represents a potential broad diagnostic tool for various stages of infection and in different animal models (cattle and mice). The NcSAG1-based ICT is a useful tool for epidemiological and surveillance purposes, while NcGRA7- and, to a lesser extent, NcGRA6-based ICTs can demonstrate early infection of *N. caninum* (acute infection in mice and sub-acute infection in cattle). This result can be exploited to discriminate between early and latent infection, which is a useful approach to control of *N. caninum* if combined with the application of appropriate hygienic control measures. Even the NLA-based ICT might be useful in reducing the hazards of *N. caninum* infection in cattle because it is potent, affordable, and easy to prepare. Our various ICTs based on different antigens will pave the way for improvement of the diagnostics for bovine neosporosis and will subsequently have a great impact on aspects of *Neospora* control. Despite our sample population revealing the efficiency of NcSAG1 and the usefulness of NcGRA7, NcGRA6, and NLA-based ICTs, further analyses are required using a higher number of positive control sera from cattle infected with *N. caninum* and closely related parasites.

## Figures and Tables

**Table 1:** Summary of enzyme-linked immunosorbent assay (ELISA) and immunochromatographic test (ICT) results.

Animal	Serum Samples (Sample Number)	Number of Positive Samples							
		NcSAG1		NcGRA7		NcGRA6		NLA	
		ELISA (IgG and/or IgM)	ICT	ELISA (IgG and/or IgM)	ICT	ELISA (IgG and/or IgM)	ICT	ELISA (IgG and/or IgM)	ICT
Mouse	2 WPI ^#^ (*n* = 5)	5	5	5	5	5	4	5	4
	4 WPI (*n* = 5)	5	5	5	0	5	0	5	3
	8 WPI (*n* = 5)	5	5	5	0	5	0	5	1
	Total positive (*n* = 15)	15	15	15	5	15	4	15	8
	Non-infected (*n* = 5)	0	0	0	0	0	0	0	0
	*T. gondii* (*n* = 3)	0	0	0	0	0	0	0	0
Cattle	4 WPI (*n* = 4)	4	4	4	3	2	3	4	4
	8 WPI (*n* = 5)	5	5	3	0	0	0	5	5
	Neosporosis (*n* = 8) *	8	8	6	5	5	0	8	8
	Total positive (*n* = 17)	17	17	13	8	7	3	17	17
	Non-infected (*n* = 6)	0	0	0	0	0	0	0	0

WPI ^#^; week post-infection. Neosporosis * refers to control-positive samples obtained from the field cattle that were confirmed via immunohistochemistry (IHC) for antigen, and indirect fluorescence antibody test (IFAT) for antibody detection.

**Table 2:** Comparison of the reactivity of field cattle sera against the ELISA and ICT using NcSAG1.

Item	NcSAG1 (*n* = 53)		
	Positive (%)	Negative (%)	CI (95%)
IgG-ELISA	19 (35.8)	34 (64.2)	23.5–50.25
IgM-ELISA	5 (9.4)	48 (90.6)	3.52–21.42
IgG or IgM-ELISA ^*^	19 (35.8)	34 (64.2)	23.5–50.25
IgG and IgM ELISA ^#^	5 (9.4)	48 (90.6)	3.52–21.42
ICT	21 (39.6)	32 (60.4)	26.76–53.98

95% CI, confidence interval. ^*^ Samples positive for one or both antibodies (IgG and/or IgM-ELISA). ^#^ Samples positive for both antibodies (IgG and IgM ELISAs).

## Data Availability

The research data can be provided upon request from the correspondence author (project supervisor).

## Supplementary Materials

The following are available online at https://www.mdpi.com/article/10.3390/microorganisms9102133/s1. Table S1: Evaluation of the ICT against IgG and IgM ELISAs using NcSAG1. Figure S1: Original and unprocessed full-length blot image of SDS-PAGE for recombinant proteins and purified IgGs. Figure S2: Pearson’s correlation coefficient of ICT results against different antibodies and ELISA OD values of experimental acute and sub-acute mouse sera.
