# Supplementary material for: Comparative Evaluation of Four Potent Neospora caninum Diagnostic Antigens Using Immunochromatographic Assay for Detection of Specific Antibody in Cattle
Source: Microorganisms. 2021 Oct 11;9(10):2133. doi: 10.3390/microorganisms9102133 (PMC8541029; doi:10.3390/microorganisms9102133)
Supplement: Supplementary file 1 [file microorganisms-09-02133-s001.zip › microorganisms-1410751-supplementary.pdf]

Supplemental information file

Table S1: Evaluation of ICT against IgG and IgM ELISAs using NcSAG1.

Diagnostic parameter*	NcSAG1 (n = 53)			
	IgG ELISA	IgM ELISA	IgG or IgM ELISA*	IgG and IgM ELISA#
Sensitivity	86.4	83.3	84.2	88.9
Specificity	93.5	64.4	93.5	85.3
Estimated prevalence (%)	41.5	11.8	38	21
Kappa value	0.8044	0.2293	0.7856	0.6376
Agreement proportion	90.6	66.7	90	86.1

*Parameters were calculated using an online statistical tool (www.vassarstats.net). The strength of agreement (kappa value) between each test and ELISA were graded as fair (0.21–0.40), moderate (0.41–0.60), and substantial (over 0.61).

* Samples positive for one or both antibodies (IgG or IgM) ELISA.

# Samples positive for both antibodies (IgG and IgM) ELISAs.

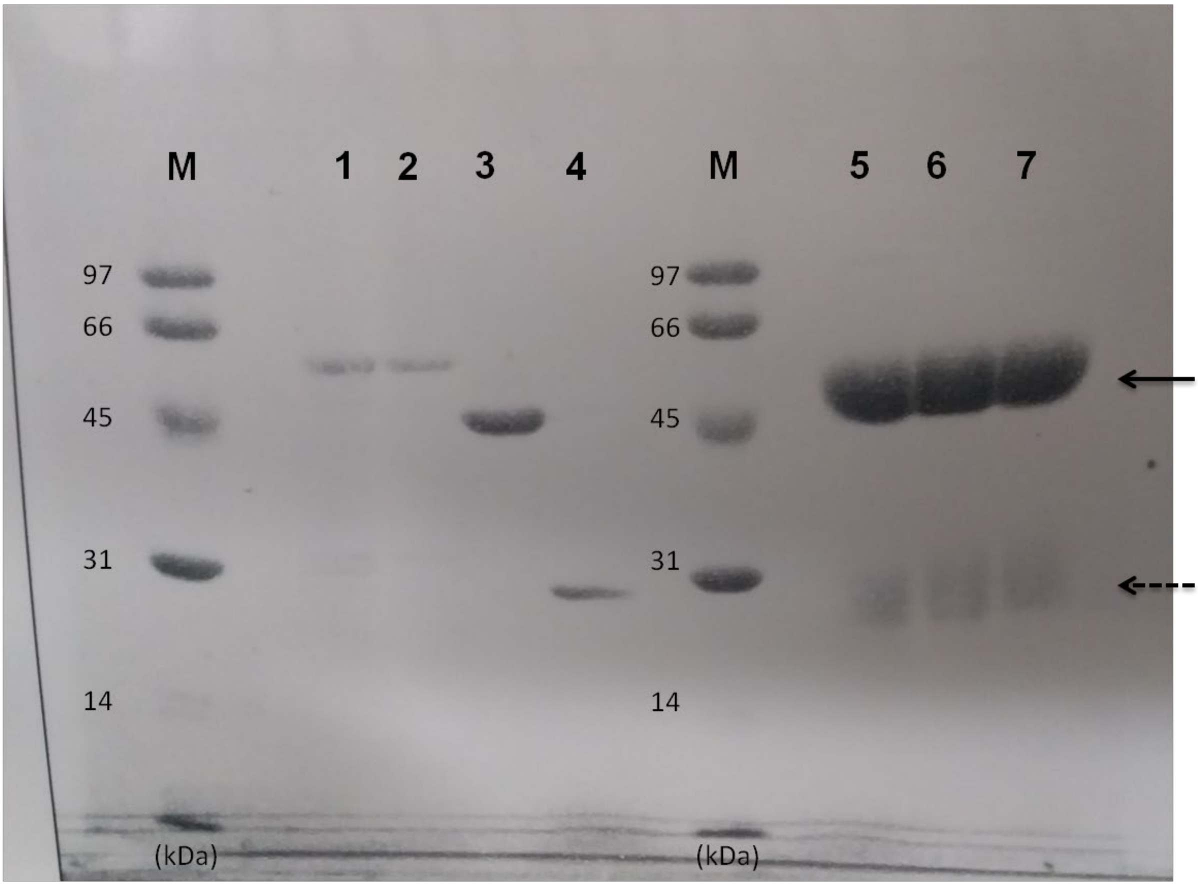

Figure S1. Original and unprocessed full-length blot image of SDS-PAGE for recombinant proteins and purified IgGs. Lanes 1-4 for recombinant proteins; lane 1, rNcSAG1+GST (57.9 kDa); lane 2, rNcGRA7+GST (59.6 kDa); lane 3, rNcGRA6+GST (50.9 kDa); lane 4, rGST (29.8 kDa); lanes 5-7 for purified rabbit IgG; lane 5, NcGRA6-antibodies; lane 6; NcGRA7-antibodies, and lane 7, NcGRA6-antibodies; lane M, low molecular weight marker. Solid black arrow refers to heavy chains and dashed black arrow refers to light chain of purified antibodies.

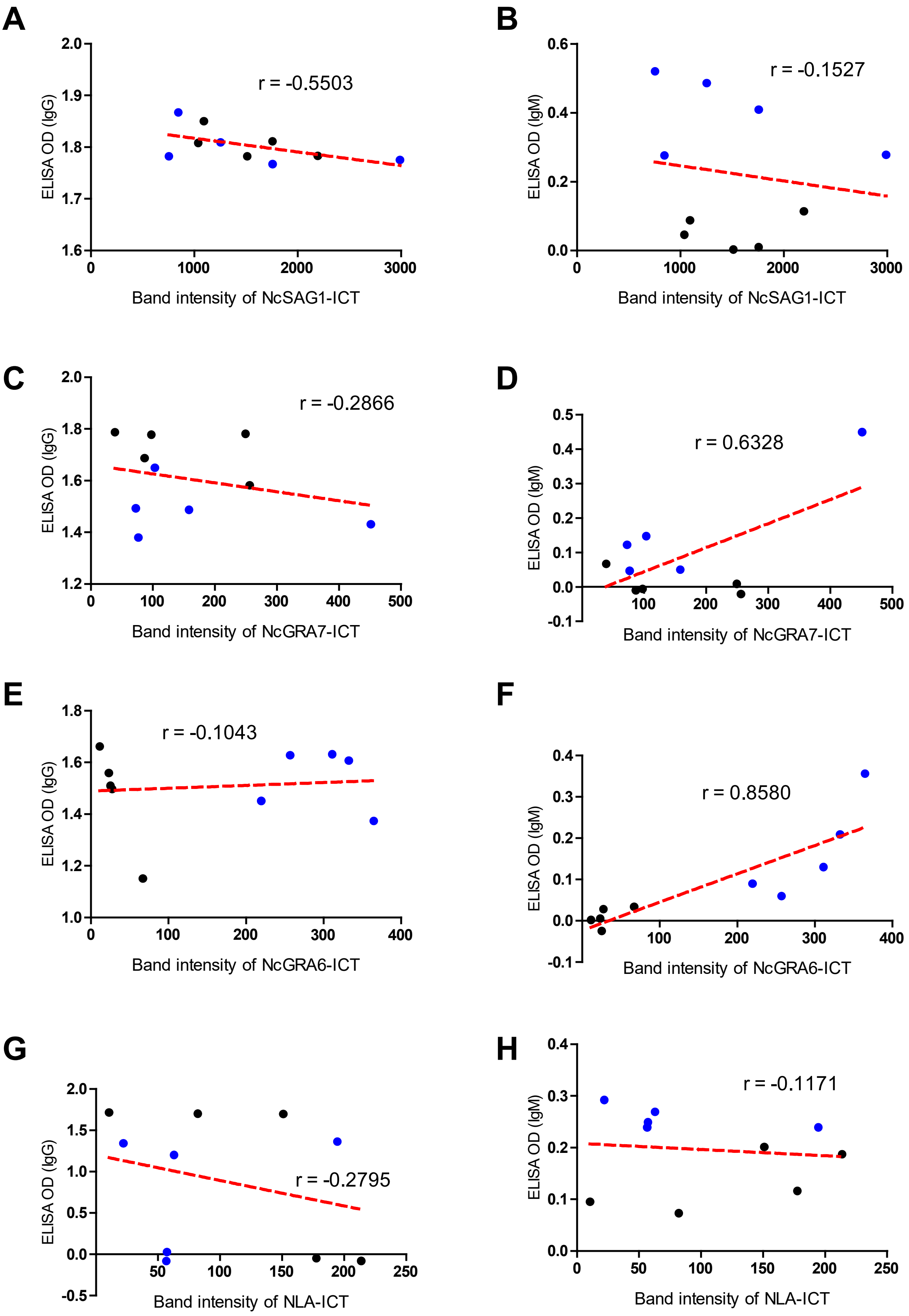
A
ELISA OD (IgG)
r = -0.5503
Band intensity of NcSAG1-ICT
B
ELISA OD (IgM)
r = -0.1527
Band intensity of NcSAG1-ICT
C
ELISA OD (IgG)
r = -0.2866
Band intensity of NcGRA7-ICT
D
ELISA OD (IgM)
r = 0.6328
Band intensity of NcGRA7-ICT
E
ELISA OD (IgG)
r = -0.1043
Band intensity of NcGRA6-ICT
F
ELISA OD (IgM)
r = 0.8580
Band intensity of NcGRA6-ICT
G
ELISA OD (IgG)
r = -0.2795
Band intensity of NLA-ICT
H
ELISA OD (IgM)
r = -0.1171
Band intensity of NLA-ICT

Figure S2. Pearson's correlation coefficient of ICT results against different antibodies and ELISA OD values of experimental acute and sub-acute mouse sera. Scatter graphs show the correlation between relative intensity of the test band in the ICT and absorbance values in the ELISA using mouse sera from acute (2 wpi; n = 5) and sub-acute (4 wpi; n = 5) phases of *N. caninum* infection. Correlation coefficients were calculated using Pearson's correlation coefficient: $|r|$ = 0.70, strong correlation; $0.5<|r|<0.7$, moderately strong correlation; and $|r|$ = 0.3–0.5 weak to moderate correlation. NcSAG1, correlation coefficient (r): r = -0.5503 for IgG-ELISA (A), and r = -0.1527 for IgM-ELISA (B). NcGRA7, r = -0.2866 for IgG-ELISA (C), and r = 0.6328 for IgM-ELISA (D). NcGRA6, r = -0.1043 for IgG-ELISA (E), and r = 0.8580 for IgM-ELISA (F). NLA, r = -0.2795 for IgG-ELISA (G), and r = -0.1171 for IgM-ELISA (H). Blue colored-dots refer to samples from 2 wpi while black-colored dots to those of 4 wpi.
